# The Reticulospinal Pathway Does Not Increase Its Contribution to the Strength of Contralesional Muscles in Stroke Survivors as Compared to Ipsilesional Side or Healthy Controls

**DOI:** 10.3389/fneur.2017.00627

**Published:** 2017-11-27

**Authors:** Sheng Li, Minal Bhadane, Fan Gao, Ping Zhou

**Affiliations:** ^1^Department of Physical Medicine and Rehabilitation, McGoven Medical School, University of Texas Health Science Center at Houston, Houston, TX, United States; ^2^TIRR Memorial Hermann Research Center, TIRR Memorial Hermann Hospital, Houston, TX, United States; ^3^The University of Texas Southwestern Medical Center, Dallas, TX, United States

**Keywords:** spasticity, reticulospinal, stroke, acoustic stimulation, strength, muscle

## Abstract

**Objective:**

Startling acoustic stimulation (SAS), via activation of reticulospinal (RS) pathways, has shown to increase muscle strength in healthy subjects. We hypothesized that, given RS hyperexcitability in stroke survivors, SAS could increase muscle strength in stroke survivors. The objective was to quantify the effect of SAS on maximal and sub-maximal voluntary elbow flexion on the contralesional (impaired) side in stroke survivors as compared to ipsilesional (non-impaired) side and healthy controls.

**Design:**

Thirteen hemiparetic stroke survivors and 12 healthy subjects volunteered for this investigation. Acoustic stimulation was given at rest, during ballistic maximal and sustained sub-maximal isometric elbow contractions using low (80 dB) and high intensity sound (105 dB). The effect of acoustic stimuli was evaluated from EMG and force recordings.

**Results:**

Prevalence of acoustic startle reflex with shorter latency in the impaired biceps was greater as compared to the response in the non-impaired side of stroke subjects and in healthy subjects. Delivery of SAS resulted in earlier initiation of elbow flexion and greater peak torque in healthy subjects and in stroke subjects with spastic hemiplegia during maximal voluntary elbow flexion tasks. During sub-maximal elbow flexion tasks, SAS-induced force responses were slightly greater on the impaired side than the non-impaired side. However, no statistically significant difference was found in SAS-induced responses between impaired and non-impaired sides at maximal and sub-maximal elbow flexion tasks.

**Conclusion:**

The findings suggest RS hyperexcitability in stroke survivors with spastic hemiplegia. The results of similar SAS-induced responses between healthy and stroke subjects indicate that RS projections via acoustic stimulation are not likely to contribute to muscle strength for stroke survivors to a significant extent.

## Introduction

Weakness for voluntary contraction is a common sequela of a hemispheric stroke. Among other clinical symptoms, weakness is a primary contributor to the overall impairment (1) and specifically toward impaired motor control (2). Damage of ipsilesional motor cortex and its descending corticospinal (CS) pathway to spinal motoneurons after stroke is presumably a primary contributor to weakness. Muscle weakness can also be attributed to altered intracortical inhibition (3, 4). A number of motor rehabilitation interventions have been used for motor recovery, such as constraint-induced movement therapy (5, 6), robotic training (7–9), and body weight-supported treadmill training (10, 11). A previous longitudinal MRI study (12) has provided evidence that recovery of locomotor function after such repetitive motor training in post-stroke hemiplegia is accompanied by increased activation in the ipsilesional motor cortex and evolution from contralesional activation to ipsilesional activation.

Both CS and reticulospinal (RS) projections contribute to the motor output. Originated from the brainstem reticular system, the RS projections can influence the CS motor output from the motor cortex. The RS system can be stimulated by acoustic stimuli via a relatively simple reflex circuit, i.e., acoustic startle reflex (ASR). The reflex circuit in humans consists of the cochlear nucleus, the caudal pontine reticular nuclei, the motoneurons of the brainstem, and the spinal motoneurons activated via the medial RS pathway (13–15). After several trials of startling acoustic stimulation (SAS), ASR responses habituate, while ensuing SAS stimulates RS projections non-reflexively (16, 17). Ensuing SAS has been shown to reduce reaction time and facilitate motor initiation in healthy subjects (18–20) and after stroke (21). It can also augment the magnitude of voluntary muscle contraction in healthy subjects and Parkinson’s patients (19, 22). Furthermore, the RS projections have been shown to compensate for damage of CS pathways for motor recovery after stroke in animal models (23–28).

The role of RS system in post-stroke motor recovery in humans is still unclear. RS hyperexcitability occurs as a result of unmasking and disinhibition effect from damage to the motor cortex and its descending CS projections in patients with spastic hemiplegia (29, 30). RS hyperexcitability contributes mainly to development of spasticity, but not to motor recovery after stroke (31, 32). However, there are reports suggesting a possible role of RS in motor recovery in stroke survivors. Integration of acoustic stimuli in the forms of rhythmic cueing or music therapy into training programs, possibly via non-reflexive stimulation of RS pathways, improves initiation and pacing of voluntary movement in stroke survivors (33–36). The results of motor improvement after such training could be attributed to repetitive training and/or acoustic stimulation. However, the role of acoustic stimulation in motor improvement can not be delineated. Furthermore, Aluru et al. (37) found that that auditory rhythmic cueing improved motor performance in stroke subjects with severe spastic paresis of wrist flexors but not in those subjects with minimal impairment or spastic co-contractions. The authors argued that auditory cueing and stimulation have different effects at different stages of post-stroke recovery via recruiting distinct neural substrates.

The purpose of the present study was to first examine whether startling acoustic stimulation (SAS) could induce greater augmentation in maximum voluntary contraction (MVC) in stroke subjects with spastic hemiplegia, since RS hyperexcitability was reported at this stage of recovery (29, 30). In clinical practice, most motor training interventions use repetitive exercises at sub-maximal levels. Therefore, we also aimed to examine whether SAS-induced force increment was greater in the spastic-paretic (impaired) side than the non-impaired side in stroke subjects at sub-maximum voluntary contraction. Accordingly, a cohort of stroke subjects with chronic spastic hemiplegia and healthy subjects received unexpected SAS in the beginning of experiments to examine the occurrence frequency of ASR at rest. Subjects then received SAS during MVC and sub-maximal elbow flexion tasks in a random order to quantify and compare the SAS-induced responses.

## Materials and Methods

### Subjects

Twelve healthy subjects (age: 25–44 years; weight: 125–205 lb; five males and seven females; and three left handed and nine right handed) volunteered for this investigation. No subject had any known history or symptoms of neuromuscular or skeletal disorders. Thirteen hemiparetic stroke survivors (age: 48–92 years; eight males and five females; eight right and five left hemiplegia; and averaged 77 months after stroke) were recruited in the experiment. Table 1 displays characteristics of the stroke subjects. Inclusion criteria were as follows: (1) ≥1 year post-stroke; (2) unilateral, single stroke (no restriction on type (ischemic or hemorrhagic) with unilateral spasticity; (3) mild-to-moderate spasticity [3 or less according to modified Ashworth scale (MAS)], note that some subjects did not have spasticity in elbow flexors but had spasticity in hand and finger flexors (not shown in the table); and (4) able to voluntarily contract impaired biceps. Exclusion criteria included patients who had (1) visual deficit and/or neglect; (2) hearing or cognitive impairment; (3) unstable medical conditions; (4) presence of contracture that would limit full elbow range of motion on the impaired side; and (5) unable to understand or follow study instructions. Written consent was obtained from all subjects for their participation in the study. This study was approved by the Committee for the Protection of Human Subjects at the University of Texas Health Science Center at Houston and TIRR Memorial Hermann Hospital.

**Table 1:** Characteristics of stroke subjects (M: male, F: female, MAS: modified Ashworth scale; ip: impaired side; nip: non-impaired; ASR: acoustic startle reflex).

ID	Gender	Age (years)	Stroke onset (months)	Affected side	MAS of elbow flexor	MVC_ip (Nm)	MVC_nip (Nm)	ASR freq (impaired) (%)	ASR freq (non-impaired (%)	ASR freq (control %)
1	M	77	52	RIGHT	1	33	37.5	0	0	
2	F	58	81	RIGHT	1+	12	38	100	33	
3	F	59	81	LEFT	1	8.2	23	100	100	
4	M	60	25	LEFT	0	10	18	0	33	
5	F	61	109	LEFT	1	3.5	32	67	0	
6	M	48	67	LEFT	0	60	85	100	100	
7	M	75	14	LEFT	1	6.4	9	33	33	
8	M	92	109	RIGHT	1	17	20	100	67	
9	M	55	157	RIGHT	1+	7	40	100	0	
10	M	63	109	RIGHT	0	34	38	100	0	
11	M	68	64	RIGHT	1+	14	50	100	0	
12	F	66	88	RIGHT	1+	22	34	0	0	
13	F	62	46	RIGHT	2	18	37	0	0	
Average		64.9	77.1			19.6	35.5	61.5	28.2	14.1

*Note: Subjects 7 & 9 were not pub*.

### Experimental Setting

**Figure 1:**
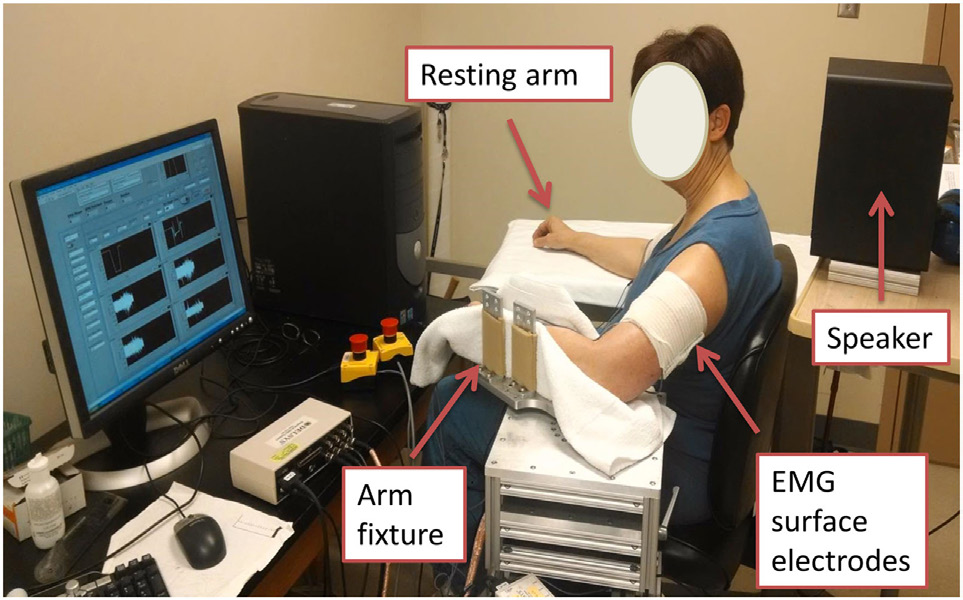
Experimental settings.

Both stroke and healthy subjects used the same experimental setup. The subjects were seated on a height adjustable chair. Conventional single differential surface electrodes (Delsys 2.1, Boston, MA, USA) were used for EMG recordings. After skin preparation, bipolar surface electromyogram (sEMG) electrodes were placed over muscle belly of biceps brachii of both dominant and non-dominant sides, according to the SENIAM recommendations (38). The electrode was secured using self-adhesive tape to ensure contact. The reference electrode of sEMG was attached to the lateral condyle of the humerus of the test arm. After placement of electrodes, the arm to be tested was firmly secured on a customized apparatus (Figure 1). The elbow joint was set to approximately 90° of flexion. The shoulder was positioned at approximately 45° of abduction and 30° of flexion. The forearm was firmly secured using four vertical plates at the proximal and distal forearm. Subjects were explicitly instructed to naturally relax their wrist and fingers, i.e., not to make a fist, or flex fingers and wrist, or to extend wrist and fingers. The center of the elbow joint was aligned with the axis of rotation of the shaft to prevent translation and rotation of the arm. The other arm of the subject was comfortably rested in an appropriately symmetrical position.

A single beep sound was generated by the computer using a 16-bit sound card (Creative Sound Blaster 16 SB1040EF) and Yamaha powered speaker (Model HS 50 M). Two levels of sound stimulus: “low” around 80 dB (for baseline response) and “high” around 105 dB (commonly used in the literature to elicit ASR) were used in this experiment. The intensity of the acoustic tone was measured and confirmed using a sound level meter (model 407730; Extech Instruments) at a distance of 30 cm from the speaker (approximately the distance to both ears of the subject).

### Experimental Tasks

The experiments consisted of the following three tasks: (1) ASR rest tasks, (2) Ballistic maximum voluntary contraction (MVC) tasks, and (3) sub-maximal elbow flexion tasks. All subjects performed ASR rest tasks first, and the order of remaining two tasks was randomized to avoid the effect of order. Healthy subjects performed on their dominant side, and stroke subjects repeated movement tasks on both sides in a random order. Thirteen stroke subjects were enrolled in the study. All of them performed the resting startle task. One stroke subject could not finish the movement tasks and one more was omitted due to motor apraxia. Only 11 of them executed ballistic tasks.

#### ASR Rest Task

Subjects were asked to relax and sound was delivered randomly between the 8th and 10th second of a 15-s trial. At first, one low sound and then three high sounds were delivered with 2-min intervals. The same order was followed for all subjects to standardize the protocol. Subjects were explicitly asked to react naturally to the sound and continue to stay relaxed as much as possible until the end of the trial. ASR responses are usually habituated after three trials, while the ensuing high sound (SAS) continues to stimulate RS tracts without causing reflex responses in healthy subjects (16, 17, 39). Only EMG signals were recorded during this task.

#### Ballistic MVC Task

Subjects performed ballistic isometric elbow contractions in response to two levels of sound stimulus at a random order. They were instructed before each trial to flex elbow joint as fast and as strong as they can, after hearing the sound and hold it for 2–3 s, i.e., a reaction time task with elbow flexion MVC. They were also instructed to focus on one object (e.g., staring at the computer screen) and be consistent throughout all trials. The sound was delivered randomly between the 4th and 8th second of a trial. Healthy subjects performed 10 trials on the dominant side, while to avoid fatigue stroke subjects performed six trials on each side. Subjects were allowed to rest between trials to minimize possible muscle fatigue. The same procedure was repeated on the other arm for stroke subjects. Before testing, subjects were required to practice the task 3–5 times before testing to familiarize themselves with the task and equipment. Similar to test trials, SAS was delivered during practice trials. The intervals between practice trials were about 30 s. As such, it was expected that practice trials were able to minimize the systematic bias, because the number of test trials was different between healthy and stroke subjects.

#### Sub-maximal Contraction Task

Subjects performed a series of isometric elbow flexion at sub-maximal levels. As in our recent study (40), elbow flexion MVC was first determined for each arm. The higher value of two MVC trials was estimated as the MVC value. Visual targets were established and displayed on the computer screen in a random order. Healthy subjects performed 3, 6, 10, 20, 30, and 40% of MVC on the dominant side. Stroke subjects performed 5, 10, 20, 30, and 40% of MVC on each side to minimize possible fatigue effect. The order of levels was randomized and balanced among subjects. Subjects were verbally cued for the beginning of a 20-s trial. Subjects then initiated elbow flexion against the vertical plates in a self-paced manner to achieve the visual target. Subjects were instructed to naturally curve the wrist and fingers during elbow flexion tasks. They were verbally encouraged to match the visual target as accurately as possible during all the trials. After the force was stabilized at the target level, high sound stimulus of 105 dB was given randomly between 6 and 8 s of the trial. Approximately 3–5 practice trials were allowed for all subjects to familiarize themselves with the task requirement. All sub-maximal isometric contractions were performed three times by healthy subjects and twice by stroke subjects to avoid fatigue. Between trials, subjects were allowed to have enough rest to minimize possible muscle fatigue.

### Data Processing and Analysis

Torque was measured with a torque sensor (Model TRS 500; Transducers Techniques, CA, USA). The output of the surface EMG electrodes was connected to an EMG amplifier (Bagnoli 8; Delsys Inc., Boston) to a PC computer with a BNC-2090A connector block and a data acquisition board (National Instruments, Austin, TX, USA). Custom LabVIEW software (National Instruments) was used. All raw sEMG and torque signals were band-pass filtered at 20–450 Hz and were digitized at 1,000 sample/s. Data were saved for offline analysis using a customized MATLAB (MathWorks, MA, USA) program. Before further processing, mean of raw EMG signal of a trial was subtracted from the EMG data to nullify any data shift. EMG signal was then rectified and filtered (Butterworth, fourth order, low cutoff 10 Hz) for finding envelop of muscle activity. Baseline activity (100 ms at the beginning of trial) was subtracted from a complete trial to nullify any data shift. Data analysis was performed by MB who performed the experiments.

#### ASR Rest Task

As described in Li et al. (30), responses to acoustic startle were quantified by (1) onset latency: time interval between the stimulus and the onset of EMG burst and (2) burst amplitude: peak amplitude of filtered EMG signals within 500 ms after the stimulus. As in previous studies (41, 42), the startle response onset was defined as time when it took the baseline EMG to increase by 2 SDs. The onset was confirmed by visual inspection and marked on the raw EMG signals from biceps muscles. Burst amplitude was computed by subtracting the baseline value (200 ms before stimulation) from mean of 10 ms data centered on the peak EMG value. Response frequency was calculated as percent of trials with ASR responses as a group (Table 2).

**Table 2:** Stroke subject acoustic startle reflex parameters: (1) onset latency (OL): time interval between the onset of stimulus and onset of EMG burst and (2) burst amplitude (BA): peak amplitude of rectified EMG.

Subj ID		Impaired side				Non-impaired side			
		T1	T2	T3	Response freq (%)	T1	T2	T3	Response freq (%)
1	OL	–	–	–	0.00	–	–	–	0
	BA	–	–	–		–	–	–	
2	OL	170	166	168	100	206	–	–	33
	BA	0.004	0.003	0.002		0.002	–	–	
3	OL	135	108	133	100	135	137	142	100
	BA	0.012	0.026	0.017		0.044	0.043	0.004	
4	OL	–	–	–	0	–	–	123	33
	BA	–	–	–		–	–	0.00	
5	OL	133	174	–	67	–	–	–	0
	BA	0.003	0.001	–		–	–	–	
6	OL	109	111	94	100	102	94	90	100
	BA	0.049	0.013	0.012		0.020	0.010	0.009	
7	OL	154	–	–	33	154	–	–	33
	BA	0.001	–	–		0.002	–	–	
8	OL	84	102	84	100	115	–	90	67
	BA	0.189	0.094	0.094		0.002	–	0.003	
9	OL	94	88	75	100	–	–	–	0
	BA	0.016	0.050	0.027		–	–	–	
10	OL	92	102	94	100	–	–	–	0
	BA	0.022	0.002	0.035		–	–	–	
11	OL	109	96	108	100	–	–	–	0
	BA	0.074	0.018	0.031		–	–	–	
12	OL	–	–	–	0	–	–	–	0
	BA	–	–	–		–	–	–	
13	OL	–	–	–	0	–	–	–	0
	BA	–	–	–		–	–	–	
Avg response freq (%)		69	62	54	62	38	15	31	28

*Freq: frequency. T1, T2, and T3 stand for trials 1, 2, and 3, respectively*.

#### Ballistic Movement Task

Reaction onset after the sound stimulation was marked by visual inspection of EMG response. Reaction time was calculated from the difference between sound stimulation and onset of biceps EMG signals. Trials with significantly late start or incomplete movement were considered as outliers and were discarded from analysis. Mean reaction time of all selected trials was calculated for each sound level. Torque data for each trial was arranged to start at reaction onset mark. A final torque profile was created by taking average of onset matched torque profiles from these trials. Peak torque was extracted from the average torque profile for each subject. Rate of force development was calculated by finding time required to reach 70% of MVC (19, 22).

#### Sub-maximal Contraction Task

Both EMG and torque responses to sound stimulation during sub-maximal contraction were quantified. The EMG response was quantified by subtracting baseline value (average over 200 ms before stimulation) from mean of 10 ms data centered on the peak EMG value. The torque response to sound stimulation was quantified by subtracting baseline value (average over 200 ms before stimulation) from the peak torque value. Average of all trials for each contraction level was calculated. The final torque response was normalized by MVC to avoid data variation as a result of anthropometry spread between subjects. Similarly, the EMG response was normalized by the corresponding EMG value obtained from the MVC task.

#### Statistical Analysis

Given large variations, descriptive statistical analyses including response frequency and paired *t*-tests were used to evaluate startle responses. For ballistic tasks, paired *t*-tests were performed to compare reaction time and peak torque between two sound levels for healthy subjects. Two-way repeated measures ANOVA was performed with the factors SIDE (x2, impaired/non-impaired side) and SOUND (x2 levels of sound stimulus) for reaction time, peak torque, and rate of force development analysis in stroke subjects. Paired and independent *t*-tests were performed to compare the percent peak torque change. To compare the torque and EMG responses in sub-maximal tasks across all the subjects, one-way repeated measures ANOVA for controls and two-way repeated measures ANOVA for stroke survivors were performed with the factors SIDE and LEVEL (x5/x6 voluntary contraction levels). Means and standard deviations are presented in the text, while means and SEs are presented in the figures. *p* < 0.05 was chosen to indicate statistically significant differences.

## Results

### ASR Rest Task

Averaged response frequency of acoustic startle response was 62% on the impaired side, 28% on the non-impaired side, and 14% for healthy subjects (Tables 1 and 2). Averaged EMG onset latency was 126 ± 30.1 ms on the impaired side, 142 ± 34.6 ms on the non-impaired side, and 137 ± 33.2 ms in the healthy subjects. The results were consistent with previous studies showing that stroke survivors were startled more frequently with shorter latency on the impaired side (30).

### Ballistic Movement Task

**Figure 2:**
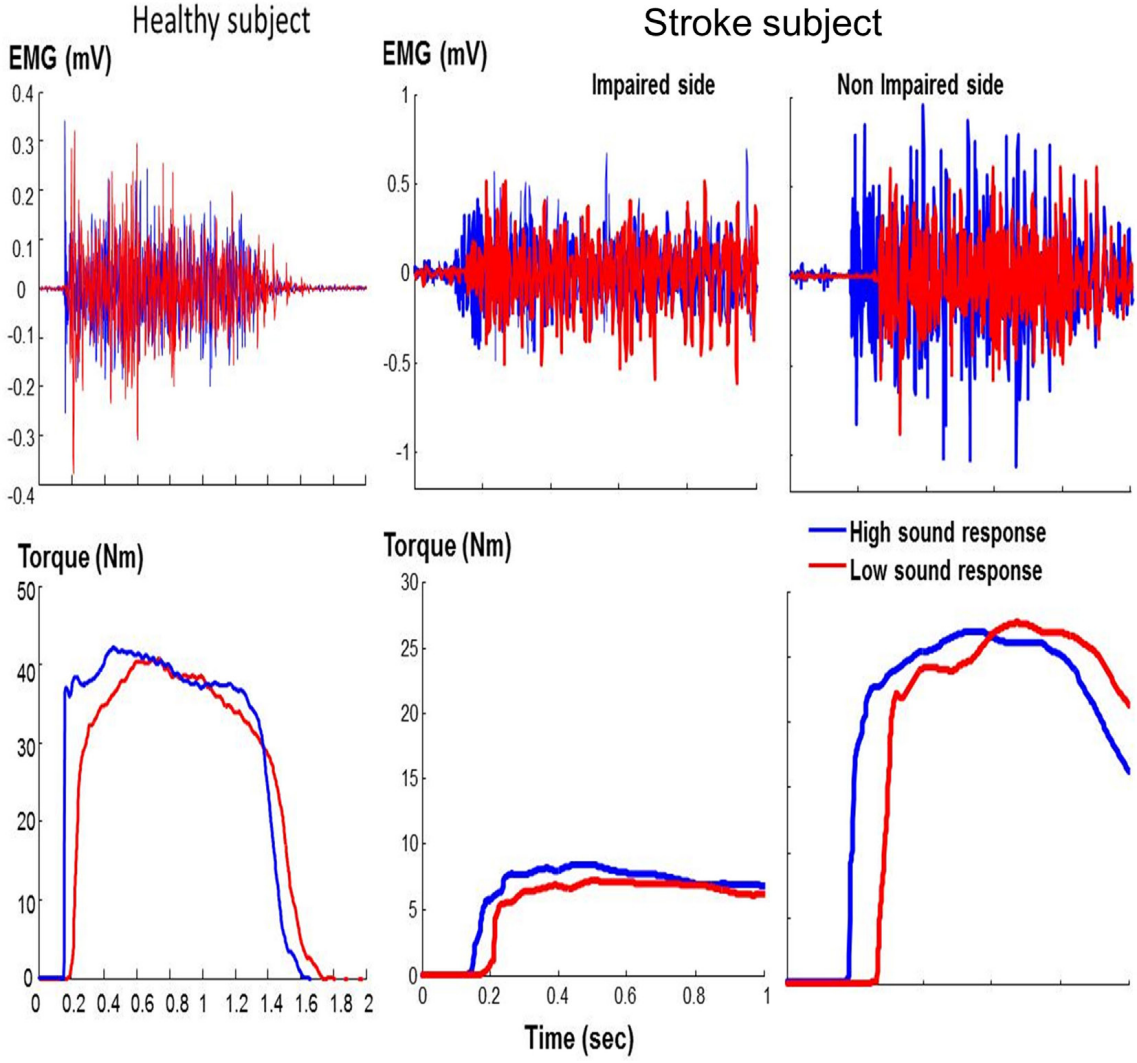
Representative trials: healthy subject and stroke subject during maximum voluntary contraction elbow flexion tasks in response to startling acoustic stimulation. Note that the *x*-axis and *y*-axis values are the same for the stroke subject.

**Figure 3:**
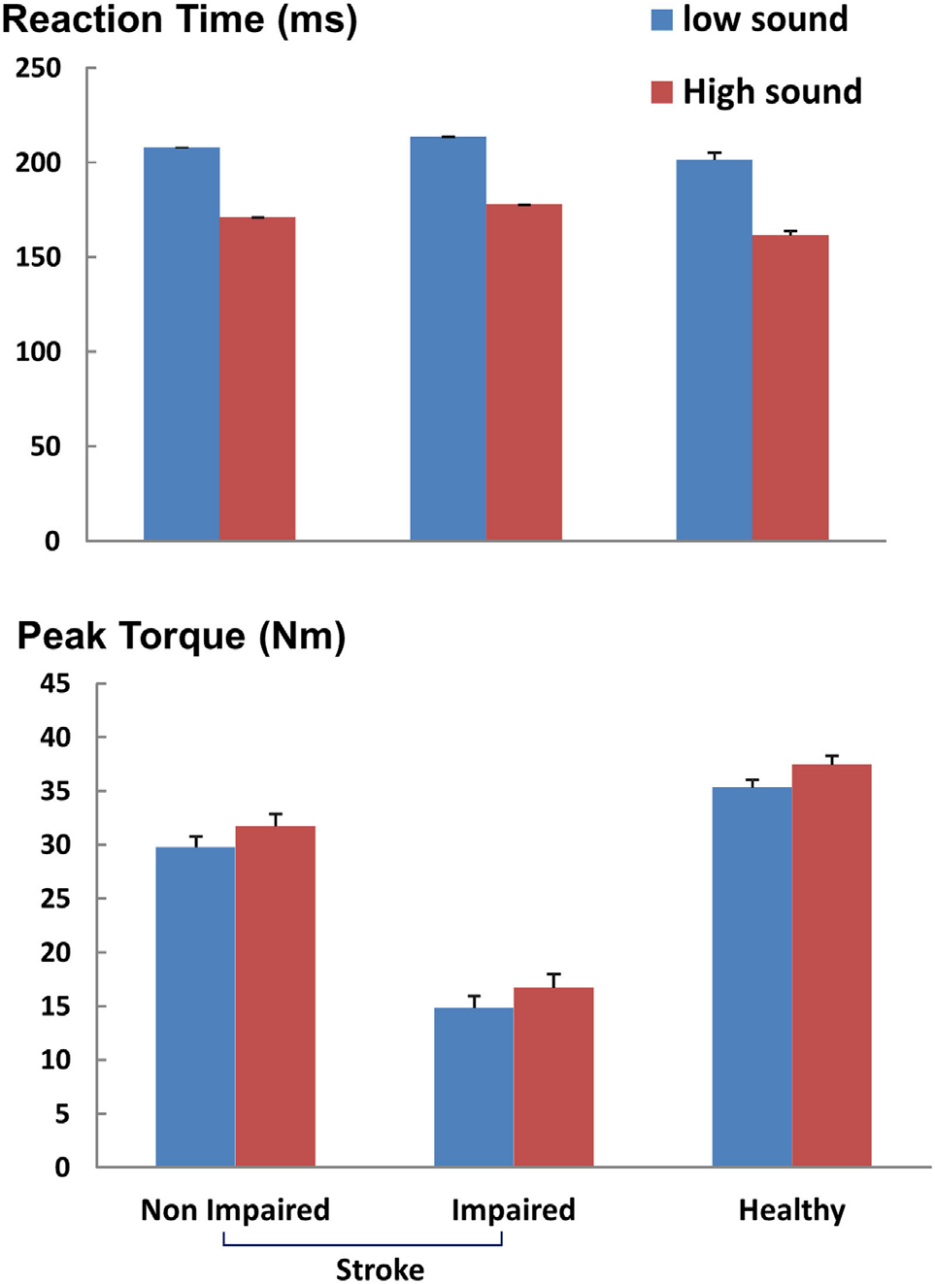
Reaction time and peak torque during ballistic tasks. ms: milliseconds, Nm: newton-meter. Mean and SEs are shown.

As shown in representative trials (Figure 2), high sound led to an early initiation of contraction and a greater peak torque in both healthy and stroke subjects. In healthy subjects, high sound stimulation significantly reduced reaction time (*p* = 0.001) and increased peak torque (*p* = 0.013) compared with low sound. The same pattern of results was observed in stroke subjects. There were main effects of SOUND on reaction time (*F*(1, 10) = 24.88, *p* = 0.0005) and on peak torque (*F*(1, 10) = 8.50, *p* = 0.0153) for both impaired and non-impaired sides (Figure 3). The percent increment – the difference between peak torque induced by low sound and high sound and then normalized to the peak torque with low sound was not significantly different between the impaired side (15.8 ± 3.1%) and the non-impaired side (8.0 ± 3.9%). The percent increment in healthy subjects (6.0 ± 2.9%) was not statistically different from those in impaired and non-impaired sides of stroke subjects. There was no significant effect on the rate of force development between impaired and non-impaired sides of stroke subjects (*F*(1, 10) = 3.8563, *p* = 0.07795).

### Sub-maximum Voluntary Contraction Task

**Figure 4:**
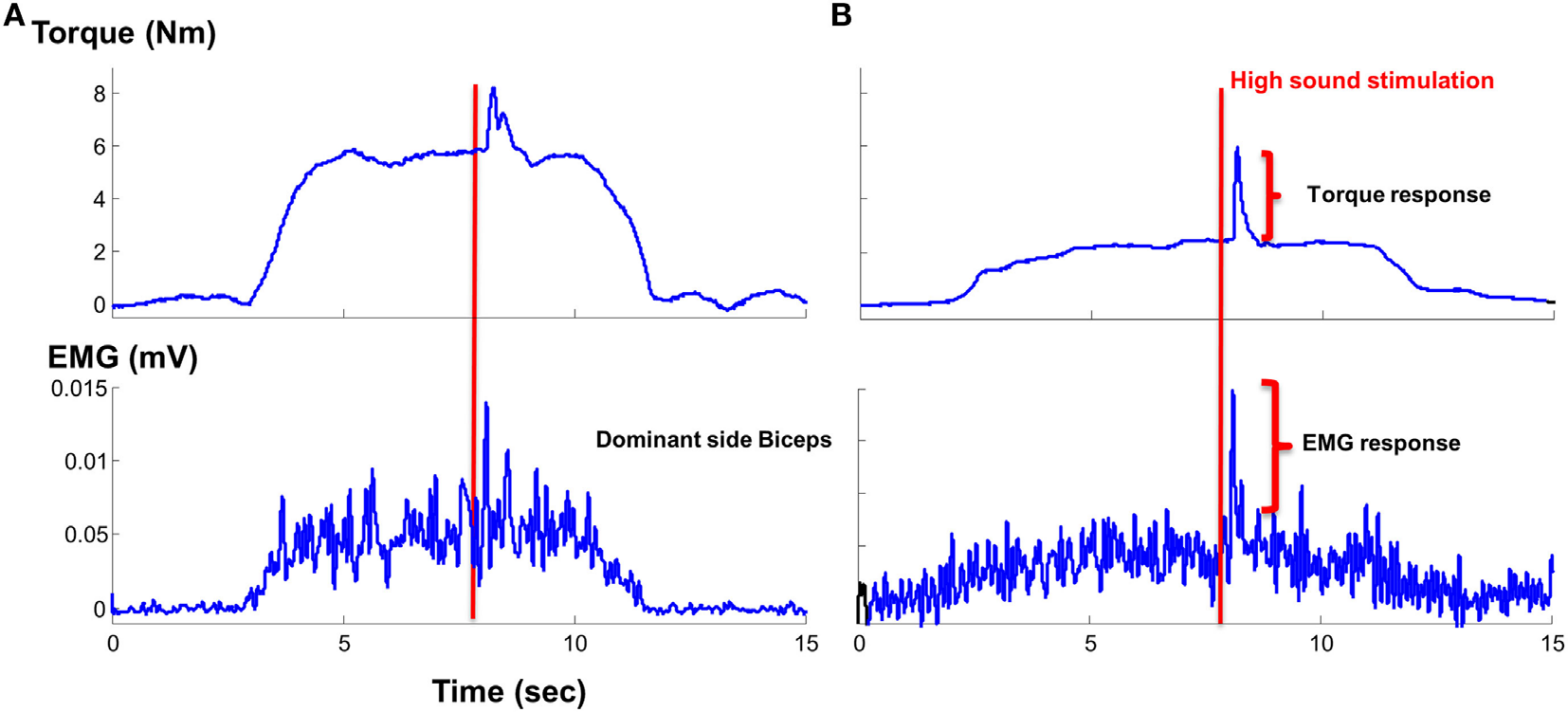
Representative trials: **(A)** healthy subject and **(B)** stroke subject during sustained sub-maximal elbow flexion tasks in response to startling acoustic stimulation.

**Figure 5:**
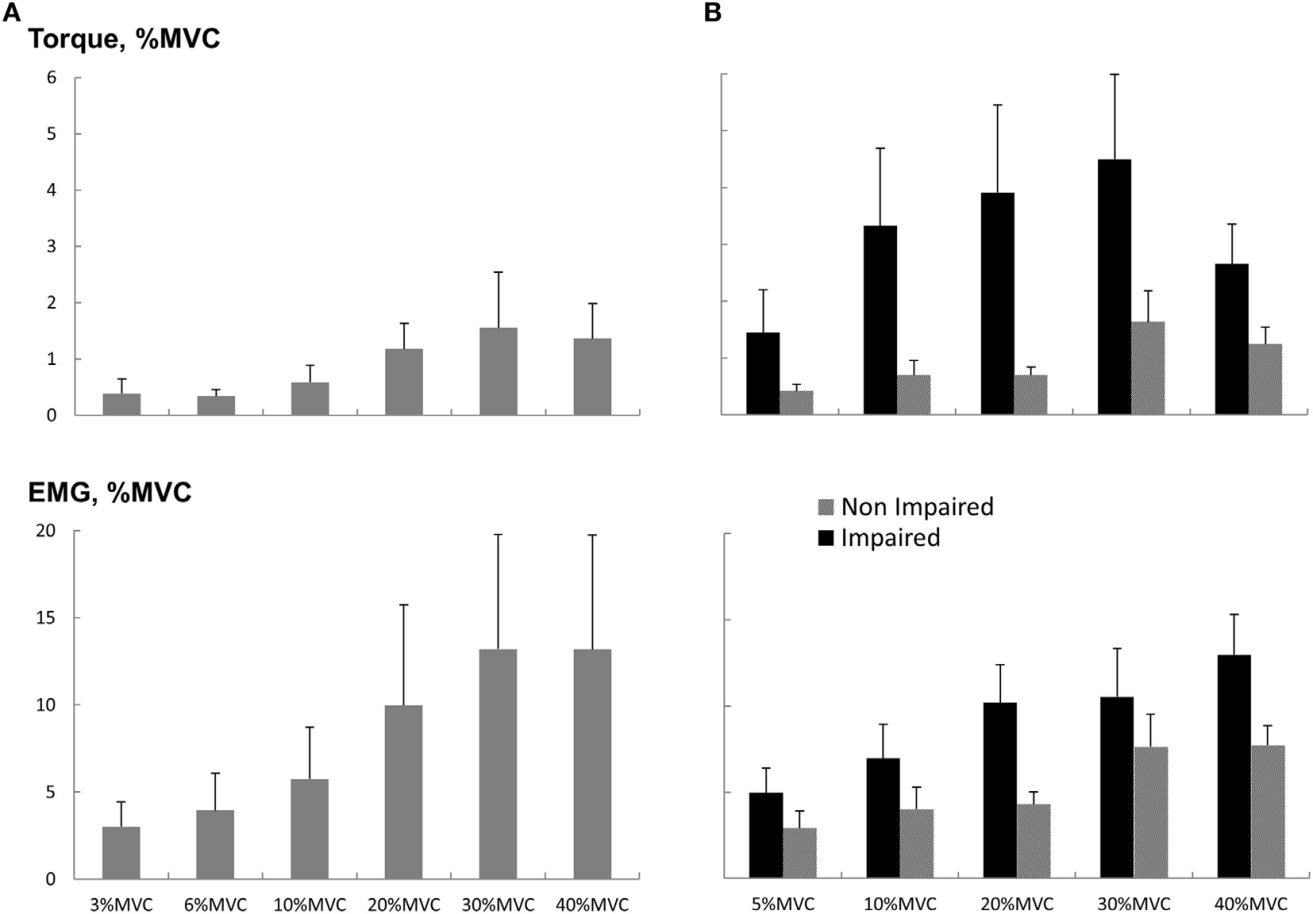
Torque and EMG response to startling acoustic stimulation during sustained sub-maximal elbow flexion. **(A)** Healthy subjects and **(B)** stroke subjects. Magnitudes were normalized to the MVC values. Means and SEs are shown. Note that the *y*-axis values for **(A,B)** are the same.

As most of the functional movements do not require maximal strength, we also evaluated the effect of loud sounds within sub-maximal contraction range. We found that while maintaining sub-maximal isometric elbow flexion, a loud sound could trigger a response in both healthy and stroke subjects (Figure 4). The torque response ranged between 0.3 and 1.5% MVC in the healthy subjects (Figure 5A). A similar range of triggered torque response (0.4–1.3% MVC) was observed on the non-impaired side of stroke subjects. In contrast, the triggered torque response was approximately 3–4 times greater on the impaired side of the stroke subjects (1.7–4.7% MVC), but with large variations (Figure 5B). There was a significant level dependence in torque response in healthy subjects (*F*(5,55) = 4.45, *p* = 0.045), but non-significant for EMG response (*F*(5,55) = 4.45, *p* = 0.52). Furthermore, a significant level dependence was observed in both torque (*F*(4, 40) = 4.4382, *p* < 0.005) and EMG response (*F*(4, 40) = 15.348, *p* < 0.001) for stroke subjects. However, no difference in normalized torque and EMG responses between impaired and non-impaired sides was found. This non-significant difference in torque response may be a consequence of normalization to MVC. We re-analyzed the SAS-induced torque responses by normalizing to the corresponding target value, i.e., percent torque increase per Nm. The same pattern of results (no statistical difference in torque response between impaired and non-impaired sides) was found. However, there was a trend toward significance (*p* = 0.08).

## Discussion

In the present study, we quantified responses to startling acoustic stimulation (SAS) from biceps brachii muscles in both healthy subjects and stroke subjects with chronic spastic hemiplegia at rest, during ballistic MVC elbow flexion and sub-maximal voluntary elbow flexion. Our results confirmed previous findings that (1) greater prevalence of ASR with shorter latency in the impaired biceps as compared to the response in the non-impaired side of stroke subjects and in healthy subjects and (2) delivery of SAS resulted in earlier initiation of elbow flexion and greater peak torque in healthy subjects. The novel findings included (1) SAS-induced reduction in reaction time and increased peak torque were also observed in stroke subjects with spastic hemiplegia during maximal voluntary elbow flexion tasks; (2) SAS-induced force responses in sub-maximal voluntary elbow flexion were similar in both healthy and stroke subjects; and (3) no statistically significant difference was found in SAS-induced responses between impaired and non-impaired sides at maximal and sub-maximal elbow flexion tasks.

Our findings of greater prevalence of ASR with shorter latency in the impaired biceps in stroke subjects with spastic hemiplegia were consistent with previous findings (29, 30). In a previous study (30), ASR occurred in 58.3% of all trials in the spastic biceps, while only 10% in the contralateral side of stroke subjects. In this study, we observed ASR responses in 61.5% of all trials in the impaired side, 28.2% in the non-impaired side of stroke subjects, and 14% in healthy subjects. These similar results support the idea of RS hyperexcitability in the impaired side of stroke survivors with spastic hemiplegia (29–32, 43).

After several trials of SAS, ASR responses habituate, while ensuing SAS stimulates RS projections non-reflexively (16, 17). In the subsequent MVC and sub-maximal elbow flexion tasks of our study, SAS stimulated RS projections non-reflexively. In MVC reaction time tasks, repetitive SAS induced earlier initiation, augmented peak force response with no difference in the rate of force development in both impaired and nonimpaired side of stroke subjects. These findings are consistent with those observed in healthy subjects and subjects with Parkinson’s disease (18–20, 44). We further observed that SAS-induced force increment during sustained elbow flexion in sub-maximal tasks in both healthy and stroke subjects. These findings extended the effect of SAS to sub-maximal levels. These findings suggest that RS stimulation via SAS could contribute and increase force output during both MVC and sub-maximal elbow flexion tasks. Given RS hyperexcitability in the impaired side of stroke subjects, the finding of statistically non-significant SAS-induced force responses between impaired and non-impaired sides of stroke subjects and healthy subjects is not trivial.

Collectively, our findings suggest that RS hyperexcitability in stroke subjects with spastic hemiplegia is not likely to contribute to development of voluntary strength in these stroke survivors to a significant extent. RS hyperexcitability represents a phenomenon of maladaptive plasticity after stroke (31, 32, 43). For those stroke involving damage to motor cortex and its descending CS projections, cortico-reticular tracts ending in lateral reticular network are usually damaged due to their anatomically proximity with CS tracts. Subsequently, function of lateral RS projections diminishes. As a result of lack of unopposed activation, medial RS hyperexcitability and its excitatory descending input to spinal reflex circuits develops. Such maladaptive RS hyperexcitability is viewed as a primary mechanism mediating post-stroke spasticity (31, 32, 43). In the course of complete motor recovery through flaccid, spastic, and recovered stages, RS hyperexcitability is only observed in the spastic stage, but normal in the flaccid or recovered stages (30). Such findings suggest that motor recovery in a late recovered stage does not rely on RS projections. As mentioned in Section “Introduction,” recovery in locomotor function is accompanied with increased ipsilesional cortical activation after repetitive motor training in stroke (12). This observation demonstrates the important role of ipsilesional motor cortex in motor recovery. Improvement in motor performance after motor rehabilitation program integrating with auditory cueing or pacing is likely to be mediated by repetitive exercise (33–36). It has been shown that intensive therapy improves motor function, but has no effect on spasticity (45). As mentioned in Section “Introduction” (37), motor training with auditory rhythmic cueing and stimulation improved motor performance in stroke subjects with severe spastic paresis of wrist flexors but not in those subjects with minimal motor impairment or spastic co-contractions. The results of different responses suggest that the outcome of motor training depends on the primary impairment. For example, if weakness is the primary impairment in subjects with severe spastic paresis, motor performance improves after increase in strength from motor training. However, no change is motor performance is expected if the primary impairment is spasticity (spastic co-contraction) or minimum weakness.

There are a number of limitations in this study. The sample sizes of healthy and stroke subjects were small. However, there were a robust pattern of findings as mentioned above (significant effects of SAS but not between two sides of stroke subjects or healthy subjects). Due to heterogeneity of stroke data, we viewed the results rather positively. Due to the experimental settings, we only recruited stroke subjects with the ability to perform voluntary elbow flexion. Stroke survivors with more severe impairment or spasticity (MAS ≥ 3) were not included. The results may not represent the features of all stroke survivors. Healthy subjects were not age matched and gender matched to stroke survivors. These factors may account for the large variations in results and may affect the results. Nevertheless, our results were in general consistent with previous findings. Future study will need to take into account these factors.

## Conclusion

In summary, we quantified and compared responses to startling acoustic stimulation (SAS) from biceps muscles in both healthy subjects and stroke subjects with chronic spastic hemiplegia at rest, during ballistic MVC elbow flexion and sub-maximal voluntary elbow flexion. Our findings of greater ASR responses in the impaired side are consistent with previous findings of RS hyperexcitability in chronic stroke with spastic hemiplegia. Our results also showed similar results of SAS-induced effects in the impaired side as compared to the non-impaired side of stroke subjects and healthy subjects. Collectively, these results suggest that RS projections via acoustic stimulation are not likely to contribute to development of voluntary strength in these stroke survivors to a significant extent.

## Ethics Statement

This study was approved by the Committee for the Protection of Human Subjects at the University of Texas Health Science Center at Houston and TIRR Memorial Hermann Hospital.

## Author Contributions

SL, MB, FG, and PZ: experimental design. MB and FG: data collection. SL, MB, FG, and PZ: study concept and design, analysis and interpretation, and critical revision of the manuscript for important intellectual content. MB and FG: acquisition of data. SL: study supervision.

## Conflict of Interest Statement

The authors declare that the research was conducted in the absence of any commercial or financial relationships that could be construed as a potential conflict of interest. The reviewer MM and handling Editor declared their shared affiliation.
